# Causal Mediation Pathways in Continuous Postprandial Glucose Monitoring for Type 1 Diabetes Patients

**DOI:** 10.21203/rs.3.rs-9100135/v1

**Published:** 2026-03-25

**Authors:** Spencer Hilligoss, Annie Qu

**Affiliations:** 1 Department of Statistics, University of California, Irvine, CA, USA.; 2 Department of Statistics and Applied Probability, University of California, Santa Barbara, CA, USA.

## Abstract

Managing postprandial glucose in Type 1 Diabetes Mellitus (T1DM) requires understanding how carbohydrate intake affects glucose through both direct pathways and insulin-mediated compensation.^1,2^ Standard analyses often treat insulin as a confounder rather than a mediator, obscuring the distinct roles of these two causal channels and hiding clinically important heterogeneity in how different patients respond to carbohydrate intake. Using meal-centered continuous glucose monitoring windows from twelve adults in the OhioT1DM 2018 and 2020 cohorts,^3,4^ we apply the causal mediation framework of Imai et al.^5^ to decompose the total effect of carbohydrate intake on glucose change into the Average Causal Mediation Effect (ACME, the indirect effect operating through insulin), the Average Direct Effect (ADE, the effect not mediated by insulin), and the Average Total Effect (ATE).^6^ We estimate these quantities by meal type over a 3.5-hour post-meal horizon and across outcome quantiles to characterize heterogeneity in glucose control mechanisms that population-average methods fail to detect.^7,8^ To adjust for confounding by longitudinal pre-meal physiological trajectories, we introduce a Causally-constrained Linear Autoencoder (CLAE) that learns low-dimensional pre-treatment representations satisfying the conditional independence assumptions required for valid mediation.^9–11^ Results reveal clinically meaningful heterogeneity in response to carbohydrate and bolus insulin intake across meal types and across the conditional glucose response distribution. At dinner, the direct glycemic effect substantially exceeds the insulin-mediated response, producing persistent total effects of 10–14 mg/dL for a +30 g carbohydrate increase that indicates systematic under-compensation by evening boluses. Breakfast, in contrast, exhibits large but nearly canceling direct and mediated effects, while lunch and snack show negligible mediation structures. Quantile-specific analysis further identifies a subgroup for whom the total carbohydrate effect at dinner reaches 22.03 mg/dL (*p* = 0.04), statistically significant despite being undetectable in the mean-level analysis. This distributional heterogeneity points to patients whose glycemic risk is undermined by population-average estimates and for whom current dosing recommendations are inadequate.^12–14^

## INTRODUCTION

Type 1 Diabetes Mellitus (T1DM) is an autoimmune condition in which pancreatic *β*-cell destruction leads to absolute insulin deficiency.^1^ Day-to-day management relies on coordinating exogenous insulin with carbohydrate intake to keep glucose within safe limits^8,15^ and to avoid acute complications such as hypoglycemia^7,16–18^ and hyperglycemia, as well as longer-term risks including neuropathy and cardiovascular disease.^19–24^ In practice, achieving stable postprandial control is difficult as patients and clinicians must align the timing of insulin action with the kinetics of glucose appearance after a meal,^25,26^ yet these processes often fail to peak together, producing early spikes or late dips.^2,27^

**Figure 1:**
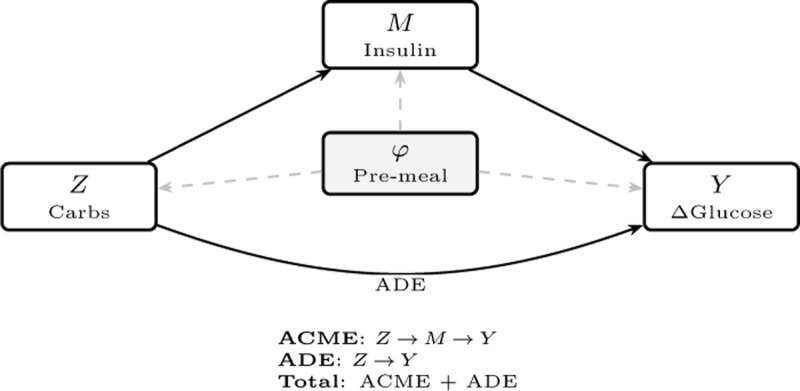
Causal mediation structure for postprandial glucose analysis. The treatment Z (meal carbohydrates in grams) affects the outcome Y (postprandial glucose change ΔG over [+60, +210] minutes) through two pathways: an indirect effect mediated by bolus insulin M (aggregated over [−120, +60] minutes) and a direct effect. Pre-treatment covariates φ, learned from the 2-hour pre-meal CGM trajectory, adjust for confounding of all three relationships (dashed arrows). The average causal mediation effect (ACME) quantifies the Z→M→Y pathway, while the average direct effect (ADE) captures carbohydrate effects not operating through insulin.

Multiple sources of variability compound this challenge. Insulin absorption and action exhibit substantial intra- and inter-individual variability.^28^ Additionally, carbohydrate counting and meal composition vary from one event to the next,^12,29^ while physiological state (sleep, activity, circadian timing) shifts across the day.^30^ Consequently, postprandial glucose responses display clear heterogeneity both between subjects and within the same subject across meals and times of day.^2,25,31,32^ Treatment sizes (both carbohydrate intake and bolus insulin dose) also vary widely, further complicating comparisons and clinical decisionmaking.^33,34^ A core inferential difficulty is that bolus insulin is typically administered in response to meal carbohydrates and thus lies on the causal pathway from carbohydrates to postprandial glucose.^35^ Figure 1 is shown for illustration. Disentangling these pathways from observational data is essential for identifying when insulin dosing is insufficient (large direct effects) versus when insulin is effective (substantial mediated effects).^36^

We address this problem using causal mediation analysis (CMA) in the counterfactual framework of Imai et al.^5,6^. Our analysis focuses on the postprandial change in glucose relative to the pre-meal baseline over a 3.5-hour post-meal horizon and decomposes effects into the average causal mediation effect (ACME), average direct effect (ADE), and total effect (ATE).^36–38^ To capture clinically relevant tail behavior, we model outcomes via quantile regression, allowing effects to vary over time and by quantile (e.g., hypoglycemia-relevant lower tail). We further characterize heterogeneity by meal type and treatment size using clinically interpretable carbohydrate contrasts anchored at the meal-type median. To improve adjustment for individual histories without hand-crafted features, we incorporate pre-treatment embeddings learned by a two-stage autoencoder trained on postprandial trajectories.^9,11^

This design targets the practical questions faced by patients and practitioners: When (over the postprandial window), for whom (across subjects and meals), and at which amount do carbohydrates exert direct effects which dominate indirect mediated effects? Our results reveal time-varying, quantile-specific patterns consistent with clinical experience, e.g., near-complete cancellation of opposing direct and mediated effects at breakfast, contrasted with persistent under-compensation by insulin at dinner, and highlight windows where carbohydrate intake strategies may be refined to better align insulin action with glucose excursions.^39,40^

## METHODS

### Dataset

We analyzed the OhioT1DM 2018 and 2020 cohorts, comprising twelve adults with type 1 diabetes monitored over eight weeks each.^3,4,41^ Participants used Medtronic 530G or 670G insulin pumps paired with Enlite CGM sensors recording interstitial glucose at 5-minute intervals.^39,42,43^ Physiological signals (heart rate, skin temperature, step counts) were collected via wrist-worn fitness bands. Meal events with carbohydrate estimates, bolus insulin deliveries, and basal rates were self-reported or logged by the pump.^29^ All analyses use de-identified data in accordance with the dataset’s license agreement. For each subject, the first 65% of days (chronologically) were assigned to training and the final 35% to testing, ensuring strict temporal separation. Standardization parameters were computed on training data only.

### Data preprocessing and defining meal-centered windows

Missing CGM values were imputed via linear interpolation when fewer than six consecutive readings were absent.^8,44,45^ For each logged meal, we constructed a window spanning [−120, 0) minutes (pre-meal) through +210 minutes (post-meal) on the 5-minute CGM grid. Windows with overlapping meals (<90 minutes apart), excessive missing data, or unresolved CGM artifacts were excluded; approximately 58 observations with post-mediator-window boluses were also excluded to preserve temporal ordering. After filtering, the analytic sample comprised 976 meal windows (777 training, 199 test). Table 1 summarizes treatment, mediator, and pre-meal glucose distributions by cohort and meal type.

### Treatment, mediator, and outcome

The treatment *Z*_*i*_ is the self-reported carbohydrate content (grams) of meal *i*.^2,12^ Treatment contrasts are defined relative to meal-type-specific medians, with the primary contrast set at +30 g. The mediator *M*_*i*_ is the total bolus insulin (units) aggregated over [−120, +60] minutes relative to the meal, capturing pre-meal, meal-time, and early correction boluses.^33,34^ This window terminates at +60 minutes to ensure the mediator is fully determined before the first outcome horizon. Approximately 12% of observations have zero bolus insulin. The outcome Δ*G*_*i*_(*t*) = *G*_*i*_(*t*) − *G*_*i*_(0) is the glucose excursion from the meal-time baseline, evaluated at *t* ∈ {60, 90, 120, 150, 180, 210} minutes post-meal.^26,46^

### Causally-constrained linear autoencoder

Causal mediation analysis requires adjustment for confounders of the treatment-mediator and mediator-outcome relationships.^5^ In our setting, these confounders are contained in longitudinal pre-meal CGM trajectories (24 × 5 = 120 features per observation), which cannot serve as covariates directly given the sample size. We developed a Causally-constrained Linear Autoencoder (CLAE) that compresses pre-meal time series into low-dimensional embeddings $\varphi \in R^{8}$ while incorporating constraints designed to satisfy the assumptions required for valid mediation analysis.^10,47^

### Encoder architecture

The encoder receives five input channels (glucose, step counts, basal insulin, meal carbohydrates, heart rate) from the [−120, 0) pre-meal window; bolus insulin is excluded to prevent mediator leakage into the learned confounders. We evaluated both convolutional (CNN) and recurrent (LSTM) encoder architectures. The CNN encoder applies successive convolutional blocks with increasing filter depth (32, 64, 128 filters) followed by global average pooling. The LSTM encoder processes the temporal sequence through a 64-unit recurrent layer. Both architectures concatenate the encoded representation with a summary of temporal feature means before a final linear projection to 8-dimensional embeddings. Categorical features (meal type, subject ID) were excluded from the encoder because they strongly correlate with treatment and caused poor balance in learned representations. Architecture details, including layer specifications and regularization parameters, are provided in Table 2.

### Training objective and causal constraints

The training objective combines two components: reconstruction losses that ensure the embeddings φ retain clinically relevant information, and causal regularization penalties that encourage the embeddings to satisfy the conditions required for valid mediation.^48^

The reconstruction component includes four prediction heads, each of which receives φ as input and predicts a different target: the pre-meal feature matrix (ensuring φ captures baseline physiological state), the insulin bolus (ensuring φ contains mediator-relevant information), and the postprandial glucose trajectory (ensuring φ is informative for the primary outcome).^49^ A fourth head predicting treatment assignment is included in the architecture for ablation purposes but is assigned zero weight in the final configuration. Assigning it positive weight would conflict with the balancing penalty described below, because an encoder that accurately predicts treatment necessarily learns representations that distinguish higher- from lower-carbohydrate meals, undermining covariate balance.^13^ Loss weights prioritize outcome prediction (weight = 2.0) over pre-treatment reconstruction and mediator prediction (each = 0.5), reflecting the relative importance of these targets for the downstream causal estimands.

The causal regularization component comprises four penalties (Table 2). The balancing penalty is the most consequential: it penalizes distributional differences in the embeddings between higher- and lower-carbohydrate meals so that the learned representation does not encode treatment information, a requirement for unconfounded estimation.^14,50^ The linearizability penalty encourages approximate linearity between φ and the outcome, ensuring compatibility with the linear models used in the mediation analysis.^38^ The conditional independence penalty targets the core identification assumption of mediation analysis (sequential ignorability; see below) by penalizing residual association between treatment and mediator after adjusting for φ.^5,51^ The stability penalty discourages sensitivity of the embedding covariance structure to resampling. Before applying these penalties, the embeddings pass through a learned nonlinear basis expansion with a residual connection, allowing the model to accommodate nonlinear relationships without requiring the user to specify a fixed functional form.^52,53^

An ablation study over five penalty configurations and both architectures, each trained with three random seeds, was used to select the final model. We prioritized covariate balance subject to adequate outcome prediction on the held-out test set (Supplementary Tables F.1 - F.3). After encoding, PCA (fit on training data only) reduces the embeddings to orthogonal components; the first 3 PCs (capturing ~90% of latent variance) serve as covariates in the mediation models.^11,54^

### Causal identification

Causal identification of mediation effects requires the sequential ignorability assumption.^5,6^ This assumption comprises two conditions. The first requires that, conditional on pre-meal state (represented by φ), the carbohydrate content of a meal is effectively randomly assigned with respect to subsequent glucose and insulin responses. The second requires that, conditional on both carbohydrate intake and pre-meal state, the insulin bolus dose is effectively randomly assigned with respect to the glucose outcome.^51^

Formally, let Y(z,m) denote the potential outcome under treatment level z and mediator level m, and let M(z) denote the potential mediator under treatment z. Sequential ignorability requires:

(1) $$\begin{array}{c} \left\{Y(z,m),M\left(z^{\prime }\right)\right\}⫫Z|X,\\ \;\text{and}\;\\ Y(z,m)⫫M|Z,X, \end{array}$$

where X denotes the principal components of φ. The CLAE architecture addresses these conditions by learning representations that minimize the residual association between treatment and mediator (via the conditional independence penalty) and that eliminate distributional differences between treatment groups (via the balancing penalty), thereby reducing the scope for violations attributable to measured confounders.

### Confounder balancing and causal mediation analysis

We estimate balancing weights using non-parametric covariate balancing propensity scores (npCBPS) for continuous treatments,^55^ regressing carbohydrate intake on the first 6 PCs of φ, glucose at meal time, and a cohort indicator. Glucose at meal time and cohort are included in the balancing model but excluded from the mediation model formulas to avoid collider bias.

For each outcome horizon, we decompose the total effect of carbohydrate intake on glucose response into the average causal mediation effect (ACME, operating through insulin), the average direct effect (ADE, all other pathways), and their sum (total effect) using the framework of Imai et al.^5,6^ Effects are estimated at three treatment contrasts: +15, +30, and +45 g of carbohydrates above the meal-type-specific median. These contrasts represent clinically interpretable increments, roughly equivalent to adding a small snack (+15 g), a slice of bread or medium fruit (+30 g), or a substantial side dish (+45 g) to a typical meal.^56^ By anchoring contrasts at the meal-type-specific median rather than a global threshold, we account for the substantial differences in typical carbohydrate intake across breakfast, lunch, dinner, and snack. The mediator model is a left-censored Tobit regression of insulin bolus on treatment and PCs to account for zero-inflation.^57^ For the outcome model, we employ two complementary specifications. The primary mean-level specification is a linear mixed-effects regression (LMER) of $\Delta G_{i}(t)$ on treatment, mediator, and PCs, with a random intercept for subject to account for within-subject correlation across repeated meal observations. To characterize distributional heterogeneity in treatment effects, we additionally estimate the outcome model via quantile regression (QR) at τ∈{0.25,0.50,0.75}, which models the conditional quantiles of the glucose response distribution rather than the conditional mean.^58,59^ Both outcome specifications are weighted by npCBPS weights and paired with the same Tobit mediator model.^14^ Mediation effects are estimated via quasi-Bayesian approximation with 1000 Monte Carlo simulations using the mediate package in R.^38,60^

### Model validation

Architecture and penalty configuration were selected on held-out test set performance, averaged across three random seeds. Embedding quality was assessed via outcome *R*^2^, mediator *R*^2^, covariate balance score, and in-range AUC for the clinically relevant 70–180 mg/dL glucose range.^8^

## RESULTS

### Model selection and representation quality

We evaluated 60 autoencoder configurations spanning two encoder architectures (CNN, LSTM), five optimizers, and six penalty regimes, each trained with three random seeds (Supplementary Table F.1). CNN architectures consistently outperformed LSTM on both outcome prediction and covariate balance; all subsequent analyses use the CNN encoder with RMSprop.

A factorial ablation^61^ of the four causal penalty components revealed that the balancing penalty is the primary driver of causal validity, improving the balance score by +0.317 at a cost of −0.071 in outcome *R*^2^ (Supplementary Table F.2). The remaining three penalties were negligible: the linearizability penalty improved outcome *R*^2^ by less than 0.01, and the conditional independence and stability penalties had similarly small marginal effects on both balance and prediction. Configurations without the balancing penalty reached *R*^2^ values as high as 0.40, but with balance scores below 0.67, indicating that the learned embeddings remained predictive of treatment, a violation of the assumptions required for valid mediation analysis. We selected the configuration using all four penalties (*R*^2^ = 0.321, balance = 0.961), prioritizing causal validity over predictive accuracy (Supplementary Table F.3).

PCA applied to the resulting 8-dimensional embeddings yielded three orthogonal components capturing approximately 90% of the latent variance, which served as covariates in all downstream models. npCBPS weighting on these components achieved adequate covariate balance, reducing mean absolute treatment correlations by 97.2% (from 0.082 to 0.002) with an effective sample size of 190 of 199 observations (95.5%), indicating that balance was achieved without reliance on extreme weights (Supplementary Table B.1). The most imbalanced covariate prior to weighting (PC_3_, *r* = −0.121) was reduced to *r* < 0.001 after weighting, and the weight distribution was well-behaved (median = 0.971, IQR = [0.91, 1.04], 1.5% exceeding 2.0). These diagnostics collectively confirm that the chosen pipeline produces embeddings that satisfy the covariate balance requirements for valid downstream causal mediation analysis.

### Pooled mediation effects

#### Direct and mediated effects at the population mean

We first present the causal mediation analysis pooled across all meal types (*N* = 190), using carbohydrate offsets of +15, +30, and +45 g from the meal-type-specific median as treatment contrasts (Supplementary Fig. D.1).

The temporal evolution of mediation effects across the postprandial window reveals a consistent pattern. The average direct effect (ADE) of a +30 g carbohydrate increase emerges at 60 minutes, rises to its peak at 120 minutes (ADE = 14.81 mg/dL, 95% CI: 5.55 to 23.65, *p* < 0.001), and remains elevated through 210 minutes. The average causal mediation effect (ACME) through insulin follows a parallel but delayed trajectory: it is non-significant at 60 minutes, becomes significant by 90 minutes, and peaks at 120 minutes (ACME = −9.64 mg/dL, 95% CI: −17.17 to −2.67, *p* = 0.01). This lag in the mediation pathway is consistent with the pharmacokinetics of rapid-acting insulin analogs, which require 60–90 minutes to reach peak action.^26^ The resulting total effect is positive but modest and generally does not reach statistical significance (total at 120 min = 5.17 mg/dL, 95% CI: −1.41 to 11.69, *p* = 0.13), reflecting the partial but incomplete cancellation of the direct glycemic impact by the compensatory insulin response.

The dose-response pattern across the +15/+30/+45 g contrasts is approximately linear (Table 3), with both ADE and ACME scaling proportionally to the treatment offset. At the +15 g contrast, the ADE at 120 minutes is 7.35 mg/dL (*p* = 0.002) and the ACME is −4.81 mg/dL (*p* = 0.01). At the +45 g contrast, these effects increase to 21.94 mg/dL (*p* = 0.004) and −14.38 mg/dL (*p* = 0.008), respectively. The proportional scaling of both pathways across dose levels supports the linearity of the underlying mediation structure and suggests that the insulin-to-carbohydrate ratio is approximately constant across the observed range of carbohydrate intake. This pattern of opposing direct and mediated effects, with partial but incomplete insulin compensation, characterizes the fundamental mediation structure across the postprandial window.

#### Quantile-specific effects at the pooled level

The mean-level results presented above characterize the average mediation structure, but the partial cancellation of opposing ADE and ACME pathways raises the question of whether this cancellation is uniform across the conditional glucose response distribution or whether it conceals subgroups for whom the net carbohydrate effect is substantially larger. We address this question using quantile regression at *τ* ∈ {0.25, 0.50, 0.75} as the outcome model, replacing the LMER specification while retaining the same Tobit mediator model and npCBPS weights.

At *τ* = 0.25, both the ADE and ACME are diminished relative to the mean-level estimates and are generally non-significant across the postprandial window (Table 4). The total effect at 120 minutes is 3.61 mg/dL (95% CI: −6.89 to 13.65, *p* = 0.52), confirming that individuals in the lower quartile of the conditional glucose response distribution experience minimal net glycemic impact from additional carbohydrate intake. At *τ* = 0.50, the mediation structure is broadly consistent with the LMER estimates: the ADE at 120 minutes is 19.71 mg/dL (*p* < 0.001) and the ACME is −10.06 mg/dL (*p* < 0.001), with a significant total effect of 9.66 mg/dL (*p* = 0.04) that already exceeds the non-significant LMER estimate at the same timepoint.

The most informative departure from the mean-level analysis occurs at *τ* = 0.75. Here, the ADE is amplified relative to the mean (19.73 mg/dL at 120 minutes, *p* = 0.02), while the ACME is attenuated (−5.79 mg/dL, *p* = 0.31). The widening gap between direct and mediated effects at this quantile produces a total effect of 13.95 mg/dL (*p* = 0.006), nearly three times the non-significant LMER total effect of 5.17 mg/dL (*p* = 0.13) at the same timepoint. This discrepancy arises because the mean averages over a distribution in which near-zero effects at lower quantiles dilute the clinically meaningful net glucose increases experienced by individuals in the upper quartile of the glucose response distribution. Depending on the starting glucose level, the *τ* = 0.75 total effect of 13.95 mg/dL can exceed the threshold commonly used as a clinically meaningful glucose excursion,^8^ indicating that the mean-level analysis fails to detect a subgroup for whom additional carbohydrate intake produces substantial and statistically significant postprandial hyperglycemia.

### Meal-type-stratified mediation analysis

The pooled mediation effects treat the carbohydrate-insulin-glucose pathway as homogeneous across the daily meal cycle. Stratified analyses reveal that this assumption is strongly violated as the mediation structure differs qualitatively across meal types, with breakfast and dinner exhibiting the most pronounced and clinically interpretable patterns. Lunch and snack show negligible mediation structure and are reported in full in Section D of the Supplementary Material.

#### Incomplete insulin compensation at dinner

Dinner (*N* = 42) shows the largest sustained mediation effects of any meal type, with both the ACME and ADE exceeding the pooled estimates in absolute magnitude (Supplementary Fig. D.3). For a +30 g carbohydrate increase, the ADE rises from 15.96 mg/dL at 60 minutes to 35.73 mg/dL at 120 minutes (*p* = 0.006) and remains above 31 mg/dL through 210 minutes. The ACME is consistently negative and significant across the full postprandial horizon, ranging from −12.09 mg/dL at 60 minutes to −22.56 mg/dL at 120 minutes (*p* = 0.01) and remaining near −22 mg/dL at 210 minutes (*p* = 0.01). Critically, unlike the near-cancellation observed at breakfast, the ADE at dinner substantially exceeds the ACME in magnitude, producing positive total effects of 10–14 mg/dL across the 90–210 minute window, though these do not reach significance (*p* = 0.14–0.30) given the smaller sample size. The sustained and large ADE at dinner suggests that evening carbohydrate metabolism produces a prolonged glycemic impact that insulin bolusing does not fully counteract.

The quantile regression analysis at dinner reveals a mediation structure that is remarkably consistent across the conditional glucose distribution, in contrast to the pooled analysis, where effects concentrate at the upper quantiles. At *τ* = 0.25, both the ADE and ACME are large in absolute magnitude (ADE = 38.28 mg/dL, *p* = 0.004; ACME = −27.49 mg/dL, *p* < 0.001), but their near-cancellation produces a non-significant total effect of 10.79 mg/dL (*p* = 0.35), indicating that individuals in the lower tail of the dinner glucose distribution still experience substantial opposing direct and mediated effects. At *τ* = 0.50, the point estimates remain large (ADE = 37.44 mg/dL, *p* = 0.06; ACME = −17.49 mg/dL, *p* = 0.18), though neither reaches significance at the 120-minute horizon. This likely reflects the wider confidence intervals associated with the smaller dinner subsample. The total effect at this quantile is 19.95 mg/dL (*p* = 0.17). At *τ* = 0.75, the total effect reaches 22.03 mg/dL at 120 minutes (*p* = 0.04), with an ADE of 49.46 mg/dL (*p* = 0.008) and ACME of −27.43 mg/dL (*p* = 0.04). This indicates that individuals in the upper tail of the dinner glucose response distribution experience net postprandial increases exceeding 20 mg/dL for a +30 g carbohydrate contrast, an effect size with clear clinical relevance for glycemic management. The consistency of the incomplete-compensation pattern from the median through the upper tail suggests that dinner bolusing is systematically miscalculated across a broad range of glucose response distributions, rather than being confined to a small subgroup of high-risk individuals (Figure 3).

#### Balanced direct and mediated effects at breakfast

Breakfast (*N* = 51) provides a revealing contrast to dinner (Supplementary Fig. D.5). For a +30 g carbohydrate increase, the ADE peaks at 90 minutes (23.46 mg/dL, *p* = 0.01) and the ACME peaks at 120 minutes (−21.15 mg/dL, *p* = 0.004). Both the direct and indirect effects are individually significant from 60 through 120 minutes (*p* < 0.05); the ACME remains significant through 180 minutes while the ADE attenuates after its 90-minute peak, and by 210 minutes both effects are non-significant (*p* > 0.10). Despite this, their near-cancellation produces total effects that are uniformly non-significant across the full horizon (all *p* > 0.35). The magnitude of both effects at their peaks, roughly double the pooled estimates, indicates that the carbohydrate-glucose and carbohydrate-insulin-glucose pathways are both amplified at breakfast relative to other meal types, yet remain sufficiently well-matched to produce net effects indistinguishable from zero. This pattern is consistent with known diurnal variation in insulin sensitivity,^62^ though the present analysis cannot determine whether the amplification reflects physiological factors such as morning insulin resistance or behavioral factors such as more consistent bolusing practices at breakfast.

The quantile regression analysis at breakfast reinforces this interpretation and provides the sharpest contrast with dinner. At *τ* = 0.25, the ADE is 17.53 mg/dL (*p* = 0.24) and the ACME is −10.28 mg/dL (*p* = 0.38); the total effect is 7.25 mg/dL (*p* = 0.23), indicating that individuals with lower glucose responses experience reduced effects through both pathways. At *τ* = 0.50, the mediation structure mirrors the LMER estimates, with an ADE of 16.40 mg/dL (*p* = 0.03) and an ACME of −17.21 mg/dL (*p* = 0.02), maintaining the near-cancellation pattern with a total effect of −0.81 mg/dL (*p* = 0.88). The most clinically informative finding at breakfast concerns the upper quantile: at *τ* = 0.75, the ACME is −24.58 mg/dL (*p* < 0.001) and the ADE is 23.58 mg/dL (*p* = 0.09), with a total effect of −1.00 mg/dL (*p* = 0.96). The near-cancellation pattern persists even at this quantile, confirming that breakfast bolusing is well-calibrated across the full glucose response distribution. This stands in direct contrast to dinner, where the gap between ADE and ACME widens at *τ* = 0.75 (Figure 3).

#### Null mediation structure at lunch and snack

Neither lunch (N=58) nor snack (N=39) exhibits a detectable mediation structure at any treatment contrast or quantile level (Supplementary Section D.4 and Section D.5). We discuss possible explanations for these null findings, including greater heterogeneity in snack bolusing behavior and more moderate insulin-to-carbohydrate coupling at midday, in the Limitations.

### Cross-meal comparison and distributional heterogeneity

**Figure 2:**
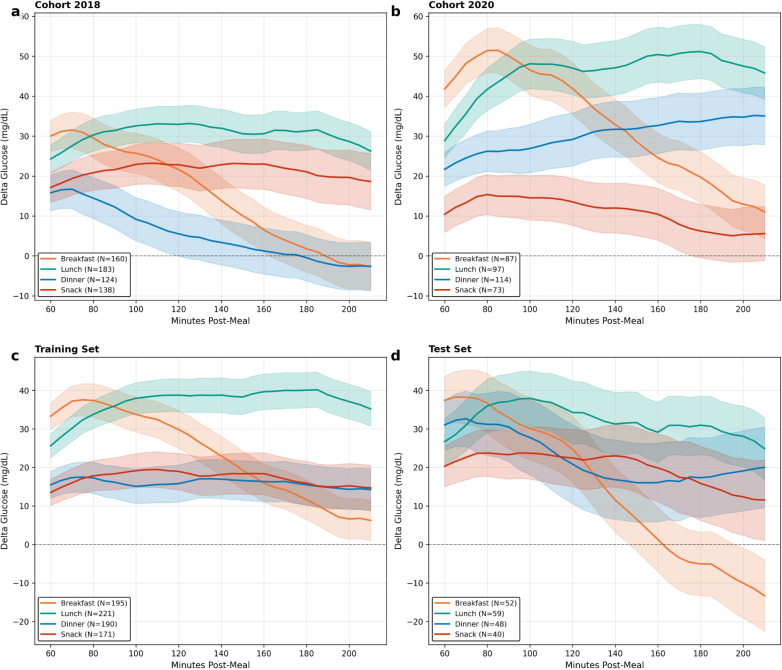
Mean postprandial delta glucose trajectories by meal type. Mean ± 1 SE glucose excursion (Δ*G*) from 60 to 210 minutes post-meal. (**a, b**) Trajectories stratified by cohort: the 2020 cohort exhibits higher and more sustained glucose excursions than 2018, particularly at lunch and dinner, reflecting between-cohort heterogeneity in postprandial glucose control. (**c, d**) Trajectories stratified by training and test sets: qualitatively similar patterns across partitions confirm that the temporal train/test split preserves the distributional characteristics of the full dataset. Across all panels, dinner (blue) and lunch (green) show the largest and most sustained excursions, while snack (red) remains near baseline, emphasizing the meal-type heterogeneity in mediation structure reported below.

Taken together, these results demonstrate that the mediation structure is not constant across the day, confirming the heterogeneity observed in the descriptive data (Table 1, Figure 2). The most notable contrast is between dinner and breakfast. At dinner, the direct effect is large and sustained, while the compensatory insulin response, though significant, is insufficient to offset it, producing the largest residual glucose impact of any meal type. At breakfast, the direct glycemic effect of additional carbohydrates is matched by a compensatory insulin response of nearly equal magnitude, yielding total effects close to zero.

The quantile regression analysis adds a distributional dimension to this cross-meal comparison, revealing that the mediation structure varies not only across meal types but also across quantiles of the conditional glucose response. At dinner, the incomplete insulin compensation is present across all quantiles as the ADE exceeds the ACME in magnitude at every quantile examined, and the total effect grows monotonically from 10.79 mg/dL at *τ* = 0.25 through 19.95 mg/dL at *τ* = 0.50 to 22.03 mg/dL (*p* = 0.04) at *τ* = 0.75. At breakfast, the near-cancellation of direct and mediated effects persists across all three quantiles examined, with the total effect remaining non-significant even at *τ* = 0.75 (−1.00 mg/dL, *p* = 0.96; Table 4). This interaction implies that distributional heterogeneity in glucose control is not a uniform scaling of mean-level effects but is concentrated at specific meal types, with dinner and the upper tail of the glucose response distribution forming the locus of greatest clinical concern.

The *τ* = 0.75 analysis identifies a subpopulation for whom the total carbohydrate effect is both large and statistically significant, despite being invisible in the mean-level analysis. For these individuals, standard insulin dosing recommendations derived from population-average effects systematically underestimate the glycemic impact of additional carbohydrates, particularly at dinner. Current automated insulin delivery algorithms rely on fixed carbohydrate-to-insulin ratios that implicitly target the average response;^63,64^ integrating quantile-aware and meal-type-specific adjustments could improve glycemic outcomes for the subpopulation most at risk of postprandial hyperglycemia.

## DISCUSSION

The central contribution of this study is the demonstration that the causal pathway from carbohydrate intake to postprandial glucose is not homogeneous. It varies qualitatively across meal types and across the conditional glucose response distribution. Population-average analyses that treat insulin as a confounder or that pool across meal types obscure this heterogeneity, yielding modest and often non-significant total effects that mask clinically important patterns operating at the meal-type and distributional levels. By decomposing the total effect into direct and insulin-mediated components and estimating these components separately by meal type and outcome quantile, the present analysis reveals structure that standard approaches fail to detect.

**Figure 3:**
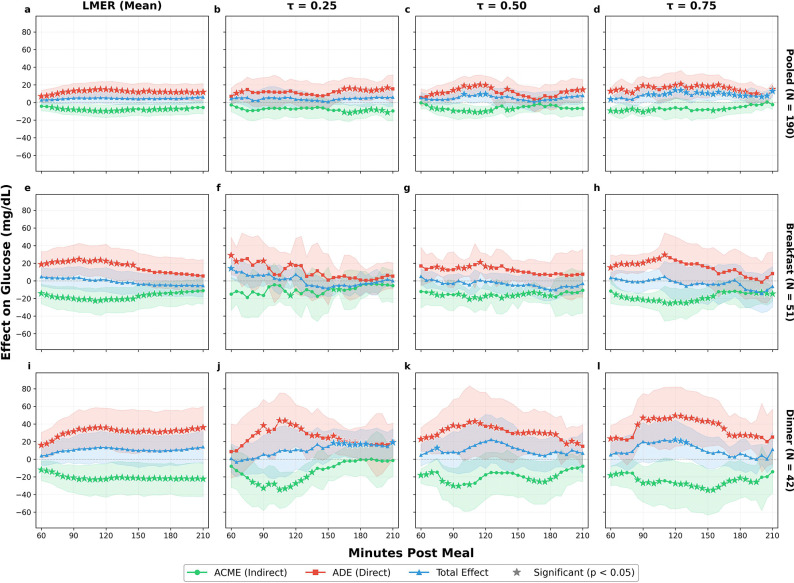
Causal mediation effects by meal type and outcome model (+30 g carbohydrate contrast). Columns show the LMER mean-level analysis and quantile regression at *τ* ∈ {0.25, 0.50, 0.75}. Rows show pooled (all meals), breakfast, and dinner analyses. (**a–d**) At the pooled level, the non-significant LMER total effect becomes significant at *τ* = 0.50 and *τ* = 0.75, revealing distributional heterogeneity masked by mean-level analysis. (**e–h**) At breakfast, the near-cancellation of ADE and ACME persists across all quantiles, indicating well-calibrated bolusing. (**i–l**) At dinner, the ADE exceeds the ACME at every quantile, with the total effect reaching significance at *τ* = 0.75 (22.03 mg/dL, *p* = 0.04). Star markers (★) denote significance (*p* < 0.05). Green = ACME (indirect effect through insulin); Red = ADE (direct effect); Blue = Total effect.

At the pooled level, the total effect of a +30 g carbohydrate increase at *τ* = 0.75 is 13.95 mg/dL (*p* = 0.006) at 120 minutes, nearly three times the non-significant mean-level estimate of 5.17 mg/dL (*p* = 0.13). This discrepancy arises because the mean averages over a distribution in which near-zero effects at lower quantiles dilute the clinically meaningful net glucose increases experienced by upper-quantile individuals. The *τ* = 0.75 quantile consistently identifies the strongest and most clinically relevant effects across meal types, corresponding to individuals in the upper quartile of the conditional glucose response distribution (Figure 3). These are the patients for whom standard dosing recommendations based on average effects are most inadequate, and for whom individualized adjustments could yield the greatest benefit.

### Clinical implications

The most actionable clinical finding concerns dinner, where both the direct and mediated effects are the largest of any meal type in absolute magnitude, but unlike breakfast, they do not cancel. The ADE exceeds the ACME throughout the postprandial window, producing total effects of 10–14 mg/dL for a +30 g carbohydrate increase. This imbalance suggests that dinner bolusing is less well-calibrated to carbohydrate load than at other meals, producing a persistent net glycemic impact. The pattern is especially pronounced at *τ* = 0.75, where the dinner total effect reaches 22.03 mg/dL at 120 minutes (*p* = 0.04), confirming that incomplete insulin compensation at dinner is a population-level phenomenon that intensifies at the upper tail. Whether this reflects suboptimal insulin-to-carbohydrate ratios at dinner, delayed carbohydrate absorption from evening meals, or reduced patient attention to bolus timing is not identifiable from these data, but the finding points to dinner as a priority target for dosing optimization.^25,33^

The clinical implication is direct: patients whose postprandial glucose responses consistently fall in the upper quartile of the conditional distribution would benefit from more aggressive dinner bolusing than what mean-level insulin-to-carbohydrate ratios prescribe. Current automated insulin delivery (AID) algorithms rely on fixed carbohydrate-to-insulin ratios that implicitly target the average response;^63,64^ integrating quantile-aware and meal-type-specific adjustments could improve glycemic outcomes for the subpopulation most at risk of postprandial hyperglycemia.^40,46,65,66^

Breakfast provides a revealing contrast. The direct and insulin-mediated effects are large, individually significant, and nearly equal in magnitude, producing total effects indistinguishable from zero. This near-complete cancellation indicates that, on average, individuals in this cohort administer bolus doses that are well-calibrated to their breakfast carbohydrate intake. The magnitude of both effects, roughly double the pooled estimates, is consistent with elevated morning insulin resistance:^62^ the same carbohydrate load produces a larger direct glycemic impact in the morning hours, but individuals compensate with correspondingly larger boluses. At *τ* = 0.75, the near-cancellation persists, reinforcing the conclusion that breakfast bolusing is well-calibrated across the glucose response distribution. From a clinical standpoint, this suggests that current breakfast bolusing practices may already be reasonably well-tuned, at least in the mean.

### Methodological considerations

The CLAE approach addresses a practical challenge in observational causal inference: how to adjust for high-dimensional pre-treatment confounders when hand-crafted feature engineering is infeasible and sample sizes are modest. Unlike standard autoencoders that optimize solely for reconstruction,^9,49,67^ the CLAE embeds the causal assumptions required for valid mediation directly into the training objective through architectural constraints and custom penalties.^10,11^

A natural concern with using learned embeddings in causal inference is interpretability. We emphasize that *φ* serves exclusively as a confounder adjustment variable, not as a quantity of direct scientific interest. The causal estimands (ACME, ADE) are defined in terms of carbohydrate intake and insulin dose, both of which remain fully interpretable on their original clinical scales. The role of *φ* is analogous to including a flexible function of baseline covariates in a propensity score model.^13^ Furthermore, because *φ* is derived exclusively from pre-treatment data (the [−120, 0) minute window), it cannot introduce post-treatment bias or encode information about the treatment, mediator, or outcome.

The ablation study provides empirical guidance on which constraints matter most. The balancing penalty was by far the most consequential, improving the covariate balance score by +0.317 at a modest cost of −0.071 in outcome *R*^2^, while the conditional independence and stability penalties contributed negligibly in marginal terms. This finding suggests that in settings where sample size constrains the number of regularization terms that can be reliably tuned, prioritizing covariate balance may be sufficient to achieve approximate causal validity.^68^ In practice, retaining three principal components from the CLAE embeddings eliminated multicollinearity, and npCBPS weighting on these components reduced the mean absolute correlation with treatment by 97.2%, with an effective sample size of 95.5%, confirming that adequate balance was achieved without reliance on extreme weights.

### Limitations

The combined 2018 and 2020 OhioT1DM cohorts^3,4^ provide rich longitudinal data but comprise a modest number of subjects, limiting generalizability to broader T1DM populations. The meal-type-stratified analyses yield sample sizes of 51 (breakfast), 58 (lunch), 42 (dinner), and 39 (snack) observations in the test set.

Assembled edited paragraph into cohesive whole Mediation was not detected at lunch or snack; full results for these meal types appear in the Supplementary Material. These null findings likely reflect substantive differences in meal-related insulin dosing behavior rather than an absence of causal structure.^69^ Snacking is inherently more heterogeneous than structured meals, with greater variability in carbohydrate content, timing, and whether a bolus is administered at all. Approximately 50% of snack boluses are missed in youth with T1DM,^70^ and unannounced snack intake degrades glucose control even under automated insulin delivery.^71^ At lunch, insulin-to-carbohydrate coupling may be more moderate and consistent than at other meals, producing neither the amplified opposing pathways seen at breakfast nor the large, incompletely offset effects seen at dinner. More broadly, breakfast, lunch, and dinner differ in their postprandial glucose profiles,^72^ with diurnal variation in insulin sensitivity further modulating the treatment-mediator-outcome relationship across meal types.^62^ Larger datasets with more subjects and greater meal-type coverage would help determine whether these explanations fully account for the null findings or whether moderate effects exist below the detection threshold of the present analysis.

### Future directions

The heterogeneity documented in this study, varying across subjects, meal types, and quantiles of the conditional glucose distribution, suggests several directions for extending the causal mediation framework to personalized diabetes management.

The most immediate extension is transfer learning.^73,74^ The per-stratum sample sizes in our meal-type analyses (42–58 test observations) limit the precision of subject-specific or meal-type-specific mediation estimates. Pre-training CLAE-type architectures on pooled or external CGM datasets and fine-tuning on individual patients or meal types would allow the estimation of personalized mediation effects that our current sample size cannot support. This strategy has proven effective in related clinical domains. Convolutional networks pre-trained on large ECG datasets achieve cardiologist-level arrhythmia detection when transferred to smaller cohorts,^75^ and deep transfer learning combined with data augmentation has been shown to improve glucose prediction in type 2 diabetes patients.^76^ Richer temporal architectures, such as the convolutional-recurrent models proposed for glucose forecasting,^77,78^ could serve as stronger approximate encoders in a transfer learning pipeline, capturing nonlinear dynamics in pre-meal trajectories that the linear CLAE encoder may not fully represent.

Data augmentation offers a complementary approach to the small-sample constraint. Augmentation strategies developed for wearable sensor time series^79^ and the theoretical analysis of linear transformations in augmentation^80^ provide a principled foundation for expanding effective sample sizes while preserving the temporal and distributional structure that causal mediation analysis requires. These techniques could be particularly valuable for the dinner and upper-quantile strata where the strongest clinical signals emerge but where observations are sparsest.

Finally, our quantile regression results highlight that the clinically critical effects concentrate in the upper tail of the glucose response distribution, a regime that is inherently underrepresented in mean-focused analyses. This connects to the broader literature on learning from imbalanced distributions,^81–83^ where standard loss functions and sampling procedures systematically underweight the tails. Integrating distributional reweighting or quantile-focused loss functions into the autoencoder training objective could improve representation quality precisely where it matters most for clinical decision-making. More broadly, the growing availability of large-scale CGM datasets^44,84^ and the maturation of machine learning methods for diabetes research create favorable conditions for scaling the causal mediation approach beyond the single-cohort analysis presented here.

## Supplementary Material

This is a list of supplementary files associated with this preprint. Click to download.

• npjMetabolicHealthandDiseaseSupplementaryMaterial.pdf

## Figures and Tables

**Table 1:** Data characteristics by cohort and meal type.

			Carbs (g)		Bolus (U)		% Zero	Glucose (mg/dL)
Cohort	Meal Type	N	Mean	SD	Mean	SD	Bolus	Mean ± SD

2018	Breakfast	160	46.5	40.4	8.10	7.05	3.8	171.7 ± 63.0
	Lunch	183	56.5	37.5	8.40	6.20	7.1	146.9 ± 52.5
	Dinner	124	62.8	44.0	10.86	7.11	7.3	157.7 ± 52.3
	Snack	138	26.6	14.7	3.88	3.52	19.6	140.2 ± 58.8

2020	Breakfast	87	47.3	24.3	5.80	4.44	14.9	143.3 ± 40.3
	Lunch	97	79.2	31.9	9.61	5.45	11.3	134.7 ± 45.3
	Dinner	114	63.8	30.0	8.33	6.45	16.7	151.1 ± 55.3
	Snack	73	27.5	18.0	4.73	4.38	21.9	149.7 ± 54.2

Summary statistics for carbohydrate intake (treatment), insulin bolus (mediator), and pre-meal glucose stratified by data collection cohort and meal category. N = number of meal observations used in the analysis; SD = standard deviation; values are mean ± SD; % Zero Bolus = percentage of meals with no insulin bolus.

**Table 2:** CLAE architecture and training configuration.

Component	Specification

*CNN Encoder (selected)*	
Conv blocks	3 blocks (32, 64, 128 filters; kernel size 3)
Pooling	Max pooling (stride 2), global average pooling
Regularization	*L_2_* = 10^−4^, dropout = 0.2, input noise *σ* = 0.05
Latent dimension	8

*Reconstruction heads*	
Pre-treatment	Predicts baseline feature matrix (weight = 0.5)
Mediator	Predicts insulin bolus (weight = 0.5)
Outcome	Predicts glucose trajectory (weight = 2.0)

*Causal penalties*	
Balancing	*γ* = 2.0
Linearizability	*λ* = 0.1
Conditional independence	*λ* = 0.05
Stability	*λ* = 0.01

*Training*	
Optimizer	AdamW (lr = 10^−3^, weight decay = 10 ^−5^)
Epochs / batch size	100 / 32
Gradient clipping	Norm ≤ 1.0

**Table 3:** PCA covariate — Pooled (all meals): LMER causal mediation effects across treatment doses (*N* = 190 meal observations, all contrast sizes).

		ACME			ADE			Total		
Time	Dose	Est.	95% CI	*p*	Est.	95% CI	*p*	Est.	95% CI	*p*

60 min	+15 *g*	−2.03	(−4.49, 0.39)	0.10	**3.41**	**(0.08, 6.38)**	**0.04**	1.38	(−0.92, 3.54)	0.22
	+30 *g*	−4.13	(−9.44, 0.67)	0.10	**6.94**	**(0.16, 13.61)**	**0.04**	2.81	(−1.77, 7.31)	0.25
	+45 *g*	−6.17	(−13.52, 0.83)	0.10	**10.24**	**(1.44, 19.11)**	**0.03**	4.07	(−2.85, 10.87)	0.25

90 min	+ 15 *g*	**−3.95**	**(−7.19, −1.01)**	**0.01**	**6.45**	**(2.29, 10.44)**	<**0.001**	2.51	(−0.37, 5.25)	0.08
	+30 *g*	**−8.10**	**(−14.08, −2.08)**	**0.01**	**13.04**	**(4.67, 21.66)**	<**0.001**	4.94	(−1.06, 10.90)	0.11
	+45 *g*	**−11.85**	**(−21.61, −2.57)**	**0.01**	**19.17**	**(6.69, 31.19)**	**0.002**	7.32	(−1.22, 16.44)	0.10

120 min	+ 15 *g*	**−4.81**	**(−8.60, −1.26)**	**0.01**	**7.35**	**(2.84, 11.89)**	**0.002**	2.54	(−0.81, 5.72)	0.13
	+30 *g*	**−9.64**	**(−17.17, −2.67)**	**0.01**	**14.81**	**(5.55, 23.65)**	**<0.001**	5.17	(−1.41, 11.69)	0.13
	+45 *g*	**−14.38**	**(−25.28, −3.96)**	**0.008**	**21.94**	**(7.91, 36.30)**	**0.004**	7.56	(−2.04, 17.19)	0.13

150 min	+ 15 *g*	**−3.75**	**(−7.07, −0.43)**	**0.04**	**5.67**	**(0.68, 10.28)**	**0.02**	1.92	(−1.38, 5.23)	0.26
	+30 *g*	−7.37	(−14.47, 0.01)	0.05	**11.39**	**(1.88, 21.76)**	**0.02**	4.01	(−2.35, 10.87)	0.21
	+45 *g*	**−11.10**	**(−21.82, −0.59)**	**0.04**	**17.11**	**(2.89, 30.89)**	**0.02**	6.01	(−3.54, 15.56)	0.22

180 min	+ 15 *g*	**−3.52**	**(−7.23, −0.04)**	**0.05**	**5.82**	**(0.98, 10.16)**	**0.01**	2.30	(−0.81, 5.43)	0.14
	+30 *g*	**−7.11**	**(−14.54, −0.20)**	**0.04**	**11.72**	**(2.62, 20.68)**	**0.02**	4.61	(−2.10, 10.82)	0.15
	+45 *g*	−10.53	(−21.01, 0.44)	0.06	**17.35**	**(3.37, 30.73)**	**0.02**	6.82	(−2.30, 16.32)	0.16

210 min	+ 15 *g*	−2.77	(−6.59, 1.15)	0.15	**5.79**	**(1.00, 10.93)**	**0.02**	3.01	(−0.48, 6.29)	0.09
	+30 *g*	−5.68	(−12.93, 1.26)	0.10	**11.77**	**(2.15, 21.45)**	**0.03**	6.09	(−0.56, 12.88)	0.07
	+45 *g*	−8.42	(−19.05, 2.03)	0.11	**17.59**	**(3.30, 31.76)**	**0.02**	9.17	(−0.85, 19.02)	0.08

Time = postprandial measurement time point in minutes after meal start. Dose = hypothetical increase in carbohydrate intake (grams) above the meal-type-specific median. ACME = Average Causal Mediation Effect (indirect effect mediated through insulin); ADE = Average Direct Effect (effect not mediated through insulin); Total = ACME + ADE. Est. = point estimate (mg/dL); 95% CI from quasi-Bayesian approximation with 1000 Monte Carlo simulations; *p* = two-sided p-value testing the null hypothesis that the effect equals zero. Significant results (*p* < 0.05) are shown in bold.

**Table 4:** Summary of mediation effects at 120 minutes post-meal (+30 g carbohydrate contrast).

		ADE		ACME		Total	
Meal	Model	Est.	*p*	Est.	*p*	Est.	*p*

Pooled	LMER	**14.81**	<**0.001**	**−9.64**	**0.01**	5.17	0.13
	τ = 0.25	10.95	0.15	−7.34	0.13	3.61	0.52
	τ = 0.50	**19.71**	<**0.001**	**−10.06**	<**0.001**	**9.66**	**0.04**
	τ = 0.75	**19.73**	**0.02**	−5.79	0.31	**13.95**	**0.006**

Breakfast	LMER	**22.38**	**0.02**	**−21.15**	**0.004**	1.23	0.85
	τ = 0.25	17.53	0.24	−10.28	0.38	7.25	0.23
	τ = 0.50	**16.40**	**0.03**	**−17.21**	**0.02**	−0.81	0.88
	τ = 0.75	23.58	0.09	**−24.58**	<**0.001**	−1.00	0.96

Dinner	LMER	**35.73**	**0.006**	**−22.56**	**0.01**	13.17	0.14
	τ = 0.25	**38.28**	**0.004**	**−27.49**	<**0.001**	10.79	0.35
	τ = 0.50	37.44	0.06	−17.49	0.18	19.95	0.17
	τ = 0.75	**49.46**	**0.008**	**−27.43**	**0.04**	**22.03**	**0.04**

All estimates are at *t* = 120 minutes post-meal for the +30 g carbohydrate contrast relative to the meal-type-specific median. Confidence intervals are 95% quasi-Bayesian from 1000 Monte Carlo simulations. Full quantile regression results for all meal types, timepoints, and dose levels appear in the Section F of the Supplementary Material.

Entries report point estimates (mg/dL) and *p*-values for the average direct effect (ADE), average causal mediation effect (ACME), and total effect at three quantiles of the conditional glucose response distribution. The LMER mean-level estimate is included for comparison. Boldface indicates statistical significance (*p* < 0.05).

## Data Availability

The data that support the findings of this study are from the 2018 and 2020 Ohio T1DM cohort^3,4^. These data are held by the dataset custodians (Ohio University) and were used under a data use agreement (DUA); therefore, restrictions apply, and the raw records are not publicly available. The data are, however, available upon request and the completion of a DUA with Ohio University.

## CODE AVAILABILITY

The code is available in a GitHub repository: https://github.com/spencha/causal002Dmediation-cgm.
